# Fish Oil and Atrial Fibrillation after Cardiac Surgery: A Meta-Analysis of Randomized Controlled Trials

**DOI:** 10.1371/journal.pone.0072913

**Published:** 2013-09-10

**Authors:** Wei Xin, Wei Wei, Zhiqin Lin, Xiaoxia Zhang, Hongxia Yang, Tao Zhang, Bin Li, Shuhua Mi

**Affiliations:** 1 Department of Cardiology, Beijing Anzhen Hospital, Capital Medical University, Beijing, China; 2 Department of Pathophysiology, School of Medicine, Nankai University, Tianjin, China; 3 Department of Cardiovascular Medicine, Affiliated Hospital of Guiyang Medical College, Guizhou, China; San Raffaele Scientific Institute, Italy

## Abstract

**Background:**

Influence of fish oil supplementation on postoperative atrial fibrillation (POAF) was inconsistent according to published clinical trials. The aim of the meta-analysis was to evaluate the effects of perioperative fish oil supplementation on the incidence of POAF after cardiac surgery.

**Methods:**

Pubmed, Embase and the Cochrane Library databases were searched. Randomized controlled trials (RCTs) assessing perioperative fish oil supplementation for patients undergoing cardiac surgery were identified. Data concerning study design, patient characteristics, and outcomes were extracted. Risk ratio (RR) and weighted mean differences (WMD) were calculated using fixed or random effects models.

**Results:**

Eight RCTs involving 2687 patients were included. Perioperative supplementation of fish oil did not significantly reduce the incidence of POAF (RR = 0.86, 95%CI 0.71 to 1.03, p = 0.11) or length of hospitalization after surgery (WMD = 0.10 days, 95% CI: 0.48 to 0.67 days, p = 0.75). Fish oil supplementation also did not affect the perioperative mortality, incidence of major bleeding or the length of stay in the intensive care unit. Meta-regression and subgroup analyses indicated mean DHA dose in the supplements may be a potential modifier for the effects of fish oil for POAF. For supplements with DHA >1 g/d, fish oil significantly reduced the incidence of POAF; while it did not for the supplements with a lower dose of DHA.

**Conclusions:**

Current evidence did not support a preventative role of fish oil for POAF. However, relative amounts of DHA and EPA in fish oil may be important for the prevention of POAF.

## Introduction

Postoperative atrial fibrillation (POAF) is the most common complication in patients undergoing cardiac surgery [1]. The incidence of POAF reported in previous studies varies between 20% and 50%, depending on the definitions and methods of detection [2], [3]. Despite the advances in surgical techniques and perioperative care, the incidence of POAF has increased continuously over the past decades, which is believed to be due to the aging of the population undergoing cardiac surgery [4]. POAF can cause significant morbidities, including hypotension, congestive heart failure, symptoms of palpitations and fatigue, and most seriously, embolic stroke [5]. Also, POAF has been shown to increase both the length of hospitalization (LOH) and total hospital costs [2], [6]. Moreover, patients with POAF tent to have higher long-term mortality [7]. Therefore, the prevention of POAF is of great importance. Although many pharmacologic interventions have been used to prevent the development of POAF, such as β-blockers, sotalol and amiodarone, all of them have limited efficacy and are not free of side effects [4], [8]. Thus, there is an urgent need to develop novel prophylactic strategies to prevent POAF and related morbidities.

Accumulating evidence from epidemiologic studies and clinical trials has indicated that fish consumption, as well as supplementation with fish oil is associated with a reduced risk of cardiovascular mortality [9], [10], [11]. Particularly, antiarrhythmia has been suggested to be an important mechanism underlying the beneficial effects of fish oil [12], [13]. Experimental studies also suggests that fish oil, which mainly consists of two categories of marine omega-3 polyunsaturated fatty acids (n-3 PUFAs) – eicosapentaenoic acid (EPA) and ducosahexaenoic acid (DHA), may exert direct or indirect antiarrhythmic action, especially in the setting of myocardial ischemia [12], [13]. However, effects of fish oil supplementation on arrhythmia from atrium, such as POAF are still unclear. Although some experimental studies support that fish oil may prevent the development of atrial fibrillation (AF) associated with heart failure [14], [15], and recent electrophysiologic studies in human also suggests that fish oil supplementation may reduce vulnerability to inducible AF [16], [17], results of prospective randomized controlled trials evaluating the effect of fish oil supplementation on POAF are generally controversial [18], [19], [20], [21], [22], [23], [24], [25]. Therefore, we performed a meta-analysis to systematically evaluate the effect of fish oil supplementation on POAF incidence after cardiac surgery.

## Methods

The primary objective of this meta-analysis is to investigate the possible role of fish oil supplementation for the prevention of POAF. In addition, some other related outcomes, including the LOH and length of stay in the intensive care unit (ICU) after surgery, perioperative mortality, and incidence of major bleeding were also evaluated. We performed this systematic review and meta-analysis according to PRISMA (Preferred Reporting Items for Systematic Reviews and Meta-Analyses) statement [26] and Cochrane Handbook guidelines [27].

### Search strategy

Pubmed (from 1950 to November, 2012), Embase (from 1966 to November, 2012) and the Cochrane Library (Cochrane Center Register of Controlled Trials) were searched for relevant records, using the terms “omega-3 fatty acids”, “n-3 fatty acids”, “fish oil”, “fish-oil”, “marine oil”, “eicosapentaenoic acid”, “EPA”, “ducosahexaenoic acid”, “DHA” paired with “atrial fibrillation”. The search was limited to studies in humans. We also analyzed reference lists of original and review articles using a manual approach. The final literature search was performed on November 25^th^, 2012.

### Study selection

According to the objective of the current meta-analysis, studies were included for analysis if they met the following criteria: 1) published as full-length article or abstract in any language; 2) reported as a prospective, randomized, and controlled trial with a parallel design (regardless of sample size); 3) included adult human subjects (≥18 years of age) who underwent a cardiac surgery and assigned to perioperative fish oil supplementation (orally or intravenously) or a control group; 4) aimed to investigate the effect of fish oil supplementation on the prevention of POAF.

### Data extraction and quality assessment

Two authors (WX and WW) independently performed the literature searching, data extraction, and quality assessment according to the inclusion criteria. Discrepancies were resolved by consensus. Extracted data include: 1) study design characteristics: randomization, allocation concealment, blinding and withdrawals/dropout; 2) patient characteristics: number, age, sex, major comorbidities, baseline parameters of echocardiogram and concurrent therapies; 3) surgery characteristics: coronary artery bypass graft (CABG) or valve surgery, off-pump or on-pump; 4) details of treatments: regimen of supplementation, dose of EPA and DHA, ratio of EPA to DHA, treatment in control groups, and treatment and follow-up duration; 5) study outcomes: incidence of POAF, LOH and length of ICU stay after surgery, incidence of major bleeding and perioperative mortality.

The quality of the studies was first judged by Jadad Score, which evaluates the quality of randomization, generation of random numbers, concealment of treatment allocation, blinding, and reporting of withdrawals [28]. Trials scored one point for each area addressed, with a possible score between 0 and 5, where 5 represented the highest level of quality. Furthermore, we also used the 6 domains of the Cochrane risk of bias tool to evaluated the quality of the included studies, which include criteria concerning sequence generation, allocation concealment, blinding of participants, personnel and outcome assessors, incomplete outcome data, selective outcome reporting and other potential threats to validity [27].

### Statistical analysis

Dichotomous data were analyzed using risk ratio (RR) with 95% confidence intervals (CI), whereas continuous variables were analyzed using weighted mean differences (WMD). Inter-study heterogeneity was formally tested using Cochrane's Q test, and significant heterogeneity was considered existing if p value was <0.10. The I^2^ statistic, which describes the percentage of total variation across studies that is due to heterogeneity rather than chance [29], was also examined, and a value of I^2^>50% indicated significant heterogeneity among the trials [30]. Pooled analyses were calculated using fixed-effect models if no significant heterogeneity was detected by Cochrane's Q test, whereas random-effect models were applied in case of significant heterogeneity across studies. If significant heterogeneity was detected among the included studies, meta-regression studies and predefined subgroup analyses were performed to explore the possible influence of patients and study characteristics (including the numbers of the patients, mean age, gender, comorbidities, concurrent medications, surgery types, total dose of fish oil, dose of EPA and DHA, POAF incidence in controls, and Jadad Scores) on the outcomes. Median values of continuous variables were used as cut-off values for grouping studies, and random-effect models were applied for the subgroup analyses. Besides, potential publication bias was assessed with Egger regression asymmetry test [31] and funnel plots; p values were two-tailed and statistical significance was set at 0.05. Meta-analysis and statistical analysis was performed with RevMan software (Version 5.1; Cochrane Collaboration, Oxford, UK) and Stata software (Version 12.0; Stata Corporation, College Station, TX).

## Results

### Search results

**Figure 1:**
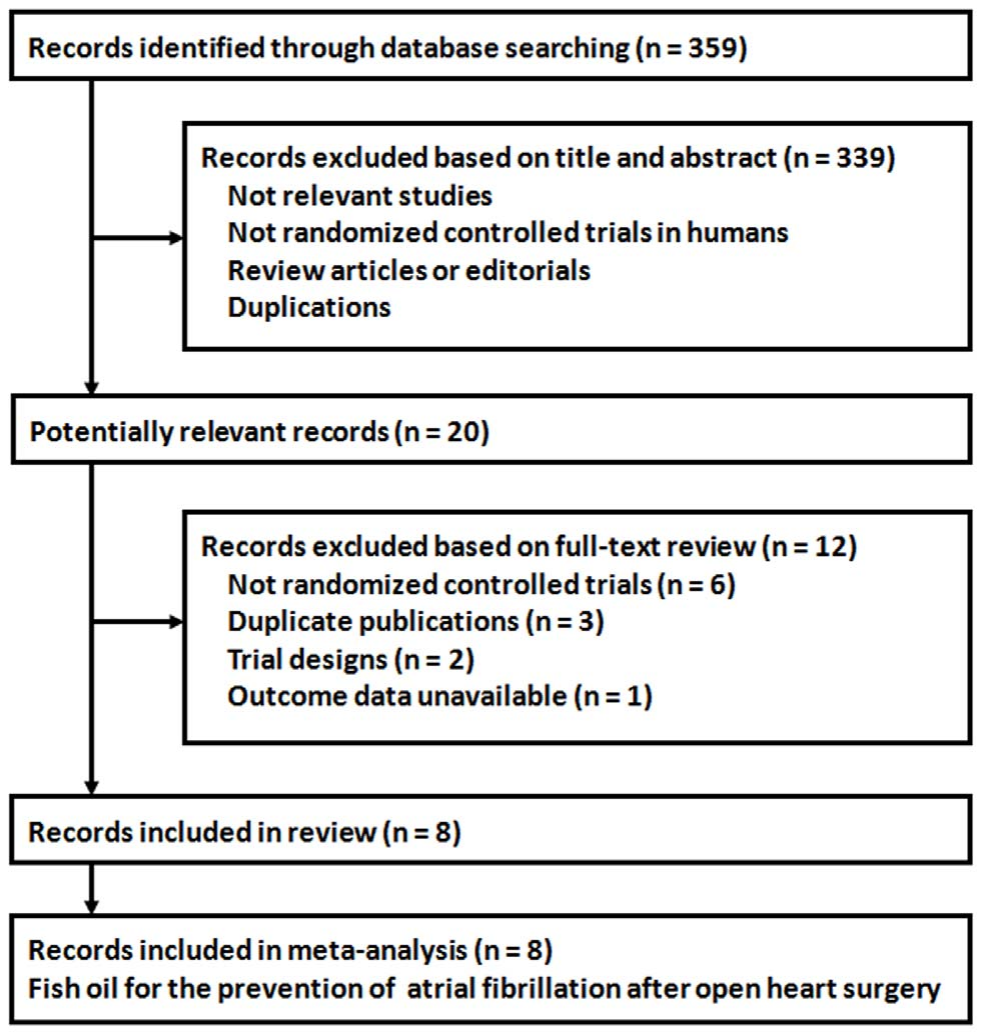
Flow diagram of the study selection procedure.

A total of 359 records were identified through the database searching, and 339 were excluded because they did not describe randomization or controlling, or because the objectives of these studies were irrelevant to the present meta-analysis, or because they were reviews, editorials or duplications. Of the 20 potentially relevant records screened, eight [18], [19], [20], [21], [22], [23], [24], [25] met the selection criteria for the current meta-analysis (**Figure** **1**). Twelve records were further excluded because six records were not randomized controlled trials; three were duplicate publications; two were trial-design papers; and one did not report available data of the related outcomes.

### Study characteristics

Overall, eight studies [18], [19], [20], [21], [22], [23], [24], [25] with 2687 patients (1337 in the fish oil group and 1350 in the control group) were included in the meta-analysis. Because the study by Sorice et al [23] includes two randomized comparisons in patients who underwent off-pump or on-pump surgery, our meta-analysis finally consists of nine sets of data comparing fish oil supplementation with controls for the prevention of POAF. The characteristics of the included studies are presented in **Table** **1** **and** **2** **.** All the studies included patients of 18 years or older, who were scheduled for cardiac surgery in the following days and of sinus rhythm on screening. Six of the studies excluded patients with previous diagnosed AF [18], , while the other two did not [24], [25]. The number of the included patients in each comparison varied from 93 to 1516, with the mean age ranging from 62.7 to 67.0 years. The concurrent medications included β-blockers, angiotensin converting enzyme inhibitors (ACEI) or angiotensin II receptor blockers (ARB), and statins in all of the studies, while one study also permitted amiodarone [25]. All of the studies enrolled patients who were scheduled for CABG, of which four studies [21], [22], [24], [25] also included patients who were admitted for valve surgery. Fish oil was supplied orally in seven studies [18], [20], [21], [22], [23], [24], [25], while the other one study [19] applied an intravenous regimen. For the two studies [24], [25] using a loading dose before surgery, we calculated the mean dose of fish oil supplied for each day according to the total amounts of supplementation and days of treatment. Overall, the mean dose of fish oil varied from 1.72 to 4.60 g/d, with the ratio of EPA to DHA varied from 0.5 to 1.4. The follow-up duration varied from during ICU stay or hospitalization to 14 days after surgery. Of note, the definition and detection of POAF varied among the individual trials as shown in **Table** **2**. None of these studies reported serious adverse events which were deemed to be caused by fish oil supplementation.

**Table 1:** Baseline characteristics of the patients of included studies.

Study	Number of patients	Mean age	Male	BMI	HTN	DM	Previous AF	CRF	COPD	Mean LVEF	Mean LAD	β-blockers	ACEI/ARB	Statins	Amiodarone	Valve surgery	Off-pump surgery
		years	%	kg/m^2^	%	%	%	%	%	%	mm	%	%	%	%	%	%
**Calo 2005**	160	65.5	85.0	NR	80.0	32.5	0	9.4	16.3	55.8	39.7	57.5	79.4	56.9	0	0	11.9
**Heidt 2009**	102	66.4	68.6	NR	NR	NR	0	NR	NR	52.2	40.3	NR	NR	NR	0	0	NR
**Saravanan 2010**	103	66.0	79.6	27.8	32.0	14.6	0	6.8	8.7	NR	NR	85.4	83.5	98.1	0	0	0
**Heidarsdottir 2010**	168	67.0	79.4	27.4	63.1	15.1	0	NR	NR	60	NR	76.2	NR	NR	0	25.6	11.9
**Farquharson 2011**	194	64.0	73.2	30.5	77.5	31.4	0	NR	10.8	64.5	NR	41.2	56.7	73	0	37.1	0.5
**Sorice 2011a** 1	93	63.5	84.9	NR	66.7	39.8	0	7.5	33.3	53	41.5	59.1	51.6	62.3	0	0	100
**Sorice 2011b** 1	108	63.0	78.7	NR	62.0	44.4	0	3.7	33.3	52.1	39.9	61.1	58.3	68.5	0	0	0
**Sandesara 2012**	243	62.7	80.7	NR	88.5	36.2	NR	2.1	14.4	52.7	39	79.8	50.6	74.1	0	11.5	25.1
**Mozaffarian 2012**	1516	63.7	72.2	28.3	75.6	26.0	7.8	6.4	11.3	56.7	42.2	57.9	51.2	56.9	3.9	49.9	11.6

1 The study by Sorice et al includes two randomized comparisons in patients who underwent off-pump or on-pump surgery. These two sets of data were analyzed separately in the meta-analysis, as Sorice 2011a and Sorice 2011b.

BMI, body mass index; HTN, hypertension; DM, diabetes mellitus; AF, atrial fibrillation; CRF, chronic renal failure; COPD, chronic obstructive pulmonary disease; LVEF, left ventricular ejection fraction; LAD, left atrial dimension; ACEI, angiotensin converting enzyme inhibitors; ARB, angiotensin II receptor blockers; NR, not reported.

**Table 2:** Characteristics of study design of included studies.

Study	Design	EPA+DHA	EPA	DHA	EPA/DHA	Control	Treatment duration	Follow-up duration	Definition of POAF	POAF detection methods	POAF incidence in controls	Jadad Score
		g/d	g/d	g/d							%	
**Calo 2005**	R, OL	Oral: 1.74	0.58	1.16	0.5	No treatment	At least 5 days before surgery until discharge	Until discharge	ECG confirmed AF (>5 min) or AF requiring intervention	Continuous monitoring at least 4 days after surgery; daily ECG	33.3	3
**Heidt 2009**	R, DB, PC	IV: 3.49	1.65	1.84	0.9	Free fatty acids	At least 0.5 day before surgery until leave ICU	Until leave ICU	ECG confirmed AF (>15 min)	Continuous monitoring and daily ECG	30.0	3
**Saravanan 2010**	R, DB, PC	Oral: 1.72	0.94	0.78	1.2	Olive oil	17 days (median) before surgery until discharge	Until discharge	ECG confirmed AF (>0.5 min)	Continuous monitoring at least 5 days after surgery; daily ECG	43.1	4
**Heidarsdottir 2010**	R, DB, PC	Oral: 2.24	1.24	1.00	1.2	Olive oil	6 days (median) before surgery until discharge or 14 days after surgery	Until discharge or 14 days after surgery	ECG confirmed AF (>5 min)	Continuous monitoring until discharge	54.1	3
**Farquharson 2011**	R, DB, PC	Oral: 4.60	2.70	1.90	1.4	Sunola	21 days before surgery until discharge or 6 days after surgery	Until discharge or 6 days after surgery	ECG confirmed AF (>10 min) or AF requiring intervention	Continuous monitoring at least 3 days after surgery; daily ECG	48.5	5
**Sorice 2011a** 1	R, OL	Oral: 1.74	0.58	1.16	0.5	No treatment	5 days before surgery until discharge	Until discharge	ECG confirmed AF (>5 min) or AF requiring intervention	Continuous monitoring at least 4 days after surgery; daily ECG	12.5	2
**Sorice 2011b** 1	R, OL	Oral: 1.74	0.58	1.16	0.5	No treatment	5 days before surgery until discharge	Until discharge	ECG confirmed AF (>5 min) or AF requiring intervention	Continuous monitoring at least 4 days after surgery; daily ECG	31.6	2
**Sandesara 2012**	R, DB, PC	Oral: 3.36(preop), 1.68(postop)	1.07	0.86	1.24	Corn oil	2.5 days(median) before surgery until 14 days after surgery	Until 14 days after surgery	ECG confirmed AF requiring intervention	Continuous monitoring and daily ECG	32.5	3
**Mozaffarian 2012**	R, DB, PC	Oral: 8.40/3∼5d or 6.72/2d (preop), 1.68(postop)	1.03	0.83	1.24	Olive oil	2–5 days before surgery until discharge or 10 days after surgery	Until discharge or 10 days after surgery	ECG confirmed AF (>0.5 min)	Continuous monitoring at least 5 days after surgery; daily ECG	30.7	5

1 The study by Sorice et al includes two randomized comparisons in patients who underwent off-pump or on-pump surgery. These two sets of data were analyzed separately in the meta-analysis, as Sorice 2011a and Sorice 2011b.

EPA, eicosapentaenoic acid; DHA, ducosahexaenoic acid; AF, atrial fibrillation; R, randomized; OL, open-label; DB, double-blind; PC, placebo-controlled; IV, intravenous; ICU, intensive care unit; ECG, electrocardiogram.

### Data quality

**Figure 2:**
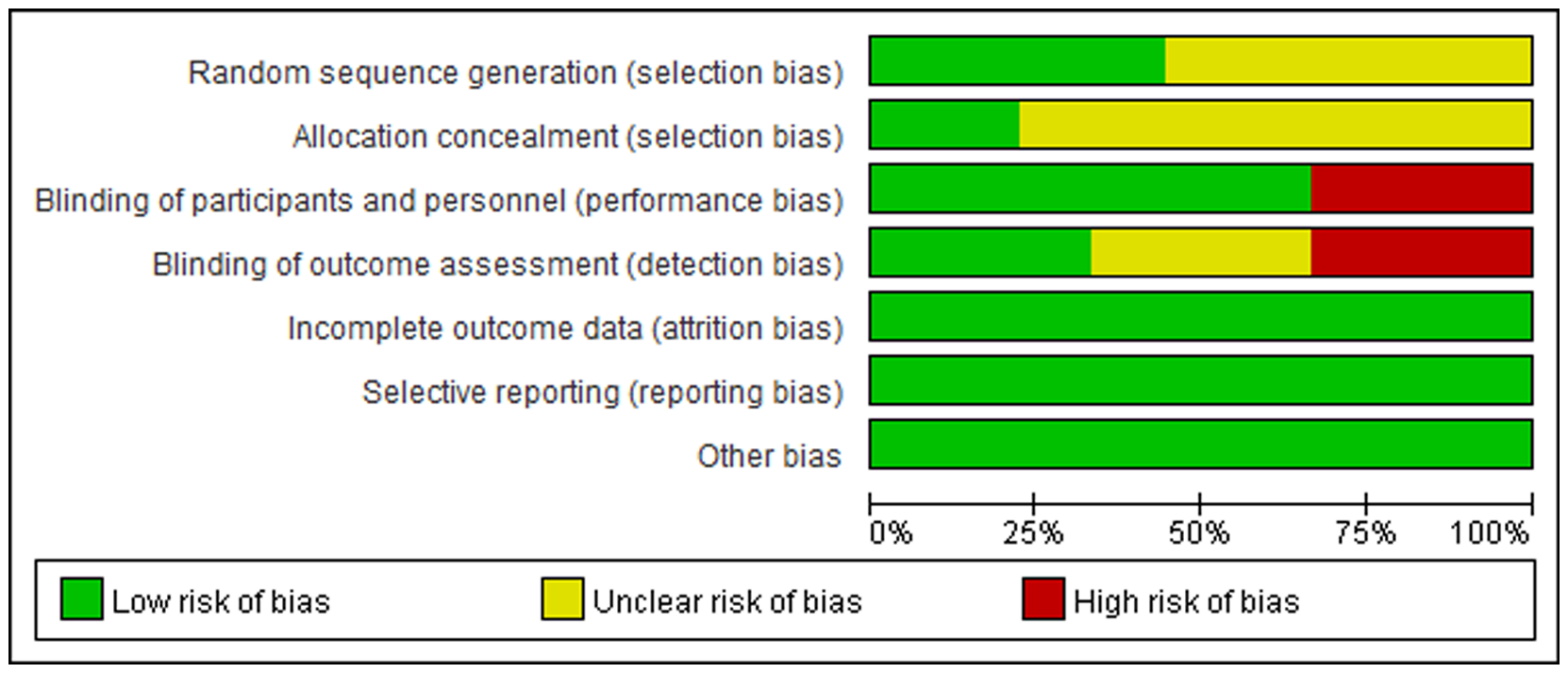
Cochrane summary risk of biases of the included studies.

The Jadad Scores of the eight studies ranged from 2 to 5. All of the included comparisons were randomized and controlled trials, with six ^8–11,13,14^ in a double-blind design and two [18], [23] of an open-label design. Four [18], [20], [22], [25] of the studies reported the methods of random sequence generation, and only two [22], [25] reported allocation concealment. Details of withdrawals and dropout were reported in all studies. The details of risks of biases of the included studies according to the Cochrane assessment tool are listed in **Table** **3** and **Figure** **2**. Two of the trials [22], [25] were at low risk of bias for all quality criteria.

**Table 3:** Cochrane risk of bias assessment.

	Sequence generation	Allocation concealment	Blinding of participants and personnel	Blinding of outcome assessment	Incomplete outcome data	Selective outcome reporting	Other potential threats
**Calo 2005**	YES	UNCLEAR	NO	NO	YES	YES	YES
**Heidt 2009**	UNCLEAR	UNCLEAR	YES	UNCLEAR	YES	YES	YES
**Saravanan 2010**	YES	UNCLEAR	YES	UNCLEAR	YES	YES	YES
**Heidarsdottir 2010**	UNCLEAR	UNCLEAR	YES	UNCLEAR	YES	YES	YES
**Farquharson 2011**	YES	YES	YES	YES	YES	YES	YES
**Sorice 2011a** 1	UNCLEAR	UNCLEAR	NO	NO	YES	YES	YES
**Sorice 2011b** 1	UNCLEAR	UNCLEAR	NO	NO	YES	YES	YES
**Sandesara 2012**	UNCLEAR	UNCLEAR	YES	YES	YES	YES	YES
**Mozaffarian 2012**	YES	YES	YES	YES	YES	YES	YES

1 The study by Sorice et al includes two randomized comparisons in patients who underwent off-pump or on-pump surgery. These two sets of data were analyzed separately in the meta-analysis, as Sorice 2011a and Sorice 2011b.

YES, low risk of bias; UNCLEAR, uncertain risk of bias; NO, high risk of bias.

### Effect of fish oil on incidence of postoperative atrial fibrillation

**Figure 3:**
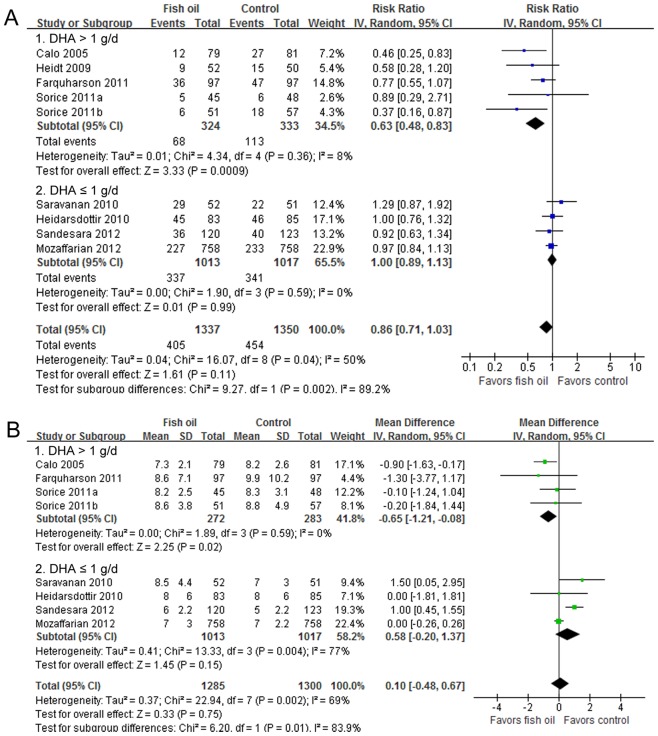
Forest plots from meta-analyses for the effects of fish oil supplementation on incidence of POAF (A) and LOH after cardiac surgery (B). The effect size of each study is proportional to the statistical weight. The diamond indicates the overall summary estimate for the analysis; the width of the diamond represents the 95% CI. POAF, postoperative atrial fibrillation; LOH, length of hospitalization; IV, inverse variance; CI, confidence interval; DHA, ducosahexaenoic acid; SD, standard deviation.

All of the nine included comparisons investigated the effect of fish oil on the incidence of POAF, and the heterogeneity among them was significant (I^2^ = 50%, p = 0.04). The pooled result with a random-effect model indicated that fish oil supplementation was not associated with a significant reduction of POAF (RR = 0.86, 95% CI 0.71 to 1.03, p = 0.11; **Figure** **3A**). The result was not significantly different when we excluded the study in which fish oil was supplied intravenously (RR = 0.88, 95% CI 0.72 to 1.06, p = 0.18).

**Figure 4:**
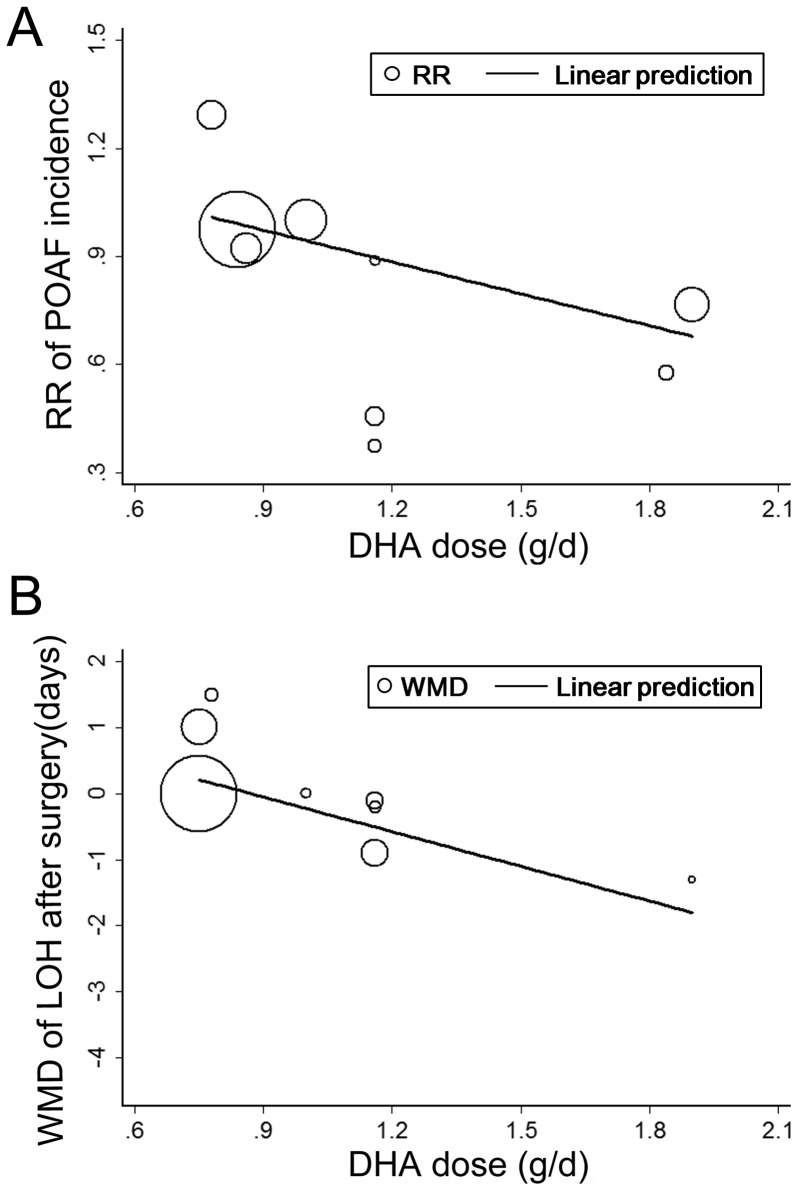
Meta-regression analyses between mean DHA dose and effects of fish oil on POAF incidence (A) and LOH after cardiac surgery (B). POAF, postoperative atrial fibrillation; LOH, length of hospitalization; DHA, ducosahexaenoic acid; RR, risk ratio; WMD, weighed mean difference.

In view of the considerable heterogeneity, we performed meta-regression analyses to explore the potential relationship between predefined study characteristics and the effect of fish oil supplementation on POAF. Results of the meta-regression tests suggested that mean dose of DHA was positively related to the preventative effect of fish oil for POAF (coefficient  = −0.29, p = 0.03; **Table** **4** and **Figure** **4A**), which could largely explain the heterogeneity. Other study characteristics, including the numbers of the patients, mean age, gender, comorbidities, concurrent medications, surgery types, total dose of fish oil, dose of EPA, POAF incidence in controls, and Jadad Scores seemed not to influence the possible effect of fish oil on POAF.

**Table 4:** Association of study characteristics and the pooled outcomes: univariate meta-regression analysis.

	RR of POAF incidence			WMD of LOH after surgery		
	Coefficient	95% CI	p	Coefficient	95% CI	p
**Number of patients**	0.00007	−0.00026 to 0.00039	0.65	−0.00006	−0.00163 to 0.00151	0.93
**Mean age (years)**	0.03	−0.09 to 0.15	0.58	−0.09	−0.66 to 0.48	0.73
**Male (%)**	0.0009	−0.0414 to 0.0431	0.96	−0.009	−0.188 to 0.170	0.91
**HTN (%)**	−0.007	−0.028 to 0.013	0.44	−0.018	−0.070 to 0.034	0.43
**DM (%)**	−0.02	−0.06 to 0.02	0.48	−0.03	−0.11 to 0.06	0.51
**CRF (%)**	−0.008	−0.178 to 0.162	0.90	−0.03	−0.09 to 0.03	0.36
**COPD (%)**	−0.03	−0.07 to 0.01	0.19	−0.02	−0.13 to 0.08	0.57
**Mean LVEF (%)**	−0.003	−0.053 to 0.046	0.86	−0.12	−0.36 to 0.12	0.25
**Mean LAD (mm)**	0.07	−0.24 to 0.38	0.68	−0.12	−1.11 to 0.87	0.72
**On β-blockers (%)**	0.007	−0.004 to 0.018	0.17	0.06	−0.12 to 0.24	0.36
**On ACEI/ARB (%)**	0.004	−0.018 to 0.025	0.69	−0.003	−0.075 to 0.070	0.93
**On statins (%)**	0.01	−0.01 to 0.03	0.26	0.05	−0.02 to 0.12	0.19
**Valvular surgery (%)**	0.001	−0.008 to 0.011	0.74	−0.005	−0.048 to 0.038	0.78
**Off-pump surgery (%)**	−0.0004	−0.0155 to 0.0148	0.96	−0.0005	−0.0272 to 0.0261	0.96
**Fish oil dose (g/d)**	−0.08	−0.24 to 0.08	0.27	0.08	−0.91 to 1.08	0.85
**EPA dose (g/d)**	−0.09	−0.36 to 0.19	0.48	−0.24	−1.98 to 1.49	0.74
**DHA dose (g/d)**	−0.29	−0.50 to −0.08	0.03	−2.1	−4.0 to −0.2	0.04
**EPA/DHA**	0.48	−0.09 to 1.05	0.08	1.17	−0.25 to 2.59	0.12
**POAF incidence in controls (%)**	0.005	−0.014 to 0.024	0.58	0.006	−0.070 to 0.081	0.86
**Jadad Score**	0.05	−0.13 to 0.24	0.52	0.01	−0.75 to 0.77	0.97

RR, relative risk; POAF, post-operative atrial fibrillation; WMD, weighed mean difference; LOH, length of hospitalization; CI, confidence interval; HTN, hypertension; DM, diabetes mellitus; CRF, chronic renal failure; COPD, chronic obstructive pulmonary disease; LVEF, left ventricular ejection fraction; LAD, left atrial dimension; ACEI, angiotensin converting enzyme inhibitors; ARB, angiotensin II receptor blockers; EPA, eicosapentaenoic acid; DHA, ducosahexaenoic acid.

Subsequent subgroup analyses also revealed that fish oil supplementation significantly reduced the incidence of POAF in patients taking DHA >1.0 g/d [18], [19], [22], [23] (RR = 0.63, 95% CI 0.48 to 0.83, p<0.001; **Figure** **3A** and **Table** **5**), while did not in those taking DHA ≤1.0 g/d [20], [21], [24], [25] (RR = 1.00, 95% CI 0.89 to 1.13, p = 0.99; **Figure** **3A** and **Table** **5**), which is consistent with the meta-regression results. Furthermore, we also found that fish oil reduced the risk of POAF more significantly among patients who were supplied with fish oil of ratio of EPA to DHA <1.2 [18], [19], [23] than those supplied with fish oil of a higher ratio [20], [21], [22], [24], [25] (p = 0.001; **Table** **5**), which further suggested that the relative amounts of DHA and EPA in fish oil supplements may influence its possible effect for POAF prevention. Besides, subgroup analyses also suggested that study design may potentially impact the effect of fish oil on POAF. Specifically, fish oil supplementation reduced the risk of POAF only in studies of an open-label design [18], [23], but did not in studies with a double-blind design [19], [20], [21], [22], [24], [25]. Of note, these open-label studies were all with a higher dose of DHA (>1.0 g/d).

**Table 5:** Subgroup analyses for the association of prespecified study characteristics and overall outcomes.

	POAF incidence						LOH after surgery				
	Studies (patients), n	I^2^	RR [95% CI]	p^1^		p^2^	Studies (patients), n	I^2^	WMD [95% CI]	p^1^	p^2^
Study design											
Double-blind studies	6 (2326)	16%	0.95 [0.83, 1.09]	0.47			5 (2224)	73%	0.44 [−0.31, 1.20]	0.25	
Non double-blind studies	3 (361)	0%	0.48 [0.31, 0.75]	0.001	0.004		3 (361)	0%	−0.61 [−1.19, −0.03]	0.04	0.03
**Number of patients**											
≤160	5 (566)	69%	0.66 [0.38, 1.15]	0.14			4 (464)	65%	−0.03 [−1.04, 0.97]	0.95	
>160	4 (2121)	0%	0.94 [0.84, 1.06]	0.34	0.22		4 (2121)	74%	0.26 [−0.54, 1.05]	0.53	0.66
**Mean age**											
<64 years	4 (1960)	38%	0.87 [0.66, 1.15]	0.33			4 (1960)	72%	0.28 [−0.38, 0.94]	0.41	
≥ 64 years	5 (727)	63%	0.83 [0.60, 1.13]	0.23	0.80		4 (625)	67%	−0.13 [−1.42, 1.16]	0.84	0.58
**Male**											
<79.5%	5 (2088)	50%	0.84 [0.68, 1.05]	0.13			4 (1986)	0%	−0.02 [−0.28, 0.24]	0.89	
≥ 79.5%	4 (599)	62%	0.87 [0.56, 1.34]	0.52	0.92		4 (599)	85*	0.32 [−0.80, 1.45]	0.57	0.56
**HTN**											
<70%	4 (472)	57%	0.92 [0.61, 1.38]	0.69			4 (472)	16%	0.30 [−0.50, 1.10]	0.46	
≥ 70%	4 (2113)	56%	0.83 [0.65, 1.05]	0.12	0.65		4 (2113)	84%	−0.05 [−0.87, 0.77]	0.90	0.54
**DM**											
<32%	4 (1981)	25%	0.97 [0.84, 1.13]	0.74			4 (1981)	41%	0.19 [−0.63, 1.01]	0.65	
≥ 32%	4 (604)	53%	0.63 [0.39, 1.02]	0.06	0.09		4 (604)	83%	−0.02 [−1.10, 1.07]	0.98	0.77
**COPD**											
<12%	3 (1813)	50%	0.97 [0.77, 1.22]	0.79			3 (1813)	61%	0.24 [−0.94, 1.42]	0.69	
≥ 12%	4 (604)	53%	0.63 [0.39, 1.02]	0.06	0.11		4 (604)	83%	−0.02 [−1.10, 1.07]	0.98	0.75
**Previous AF**											
ncluded	2 (1795)	0%	0.97 [0.84, 1.11]	0.64			2 (1759)	90%	0.47 [−0.51, 1.45]	0.35	
Excluded	7 (928)	60%	0.77 [0.57, 1.04]	0.09	0.18		6 (826)	46%	−0.16 [−0.94, 0.61]	0.68	0.32
**Baseline LVEF**											
<55%	4 (546)	32%	0.70 [0.46, 1.06]	0.09			3 (444)	52%	0.43 [−0.43, 1.30]	0.33	
≥ 55%	4 (2038)	59%	0.85 [0.68, 1.07]	0.18	0.41		4 (2038)	50%	−0.37 [−1.01, 0.27]	0.26	0.14
**Baseline LAD**											
<40mm	3 (511)	68%	0.58 [0.32, 1.06]	0.08			3 (511)	88%	−0.00 [−1.43, 1.43]	1.00	
≥ 40mm	3 (1711)	0%	0.95 [0.82, 1.10]	0.51	0.12		2 (1609)	0%	−0.01 [−0.26, 0.25]	0.97	1.00
**Valvular surgery**											
Included	4 (2121)	0%	0.94 [0.84, 1.06]	0.34			4 (2121)	74%	0.26 [−0.54, 1.05]	0.53	
Excluded	5 (566)	69%	0.66 [0.38, 1.15]	0.14	0.22		4 (464)	65%	−0.03 [−1.04, 0.97]	0.95	0.66
**Off-pump surgery**											
Included	6 (2282)	34%	0.88 [0.73, 1.07]	0.19			5 (2180)	78%	0.04 [−0.62, 0.69]	0.92	
Excluded	3 (405)	76%	0.79 [0.45, 1.38]	0.41	0.73		3 (405)	56%	0.21 [−1.36, 1.78]	0.79	0.84
**Beta-blockers used**											
<70%	4 (1963)	56%	0.79 [0.58, 1.07]	0.13			4 (1963)	50%	−0.34 [−0.92, 0.24]	0.25	
≥ 70%	4 (622)	57%	0.94 [0.69, 1.29]	0.71	0.43		4 (622)	13%	0.84 [0.25, 1.42]	0.005	0.005
**ACEI/ARB used**											
<55%	3 (1852)	0%	0.97 [0.84, 1.11]	0.62			3 (1852)	81%	0.34 [−0.42, 1.09]	0.38	
≥ 55%	4 (565)	75%	0.70 [0.42, 1.15]	0.15	0.22		4 (565)	66%	−0.18 [−1.42, 1.07]	0.78	0.49
**Statins used**											
<70%	4 (1877)	70%	0.65 [0.37, 1.12]	0.12			4 (1877)	42%	−0.27 [−0.76, 0.23]	0.29	
≥70%	3 (540)	49%	0.96 [0.71, 1.29]	0.76	0.22		3 (540)	47%	0.82 [−0.21, 1.86]	0.12	0.06
**Amiodarone used**											
Included	1 (1516)	–	0.97 [0.84, 1.13]	0.74			1 (1516)	–	0.00 [−0.26, 0.26]	1.00	
Excluded	8 (1171)	53%	0.81 [0.63, 1.03]	0.09	0.20		7 (1069)	72%	0.10 [−0.74, 0.93]	0.82	0.83
**Fish oil regimen**											
Intravenously supplied	1 (102)	–	0.58 [0.28, 1.20]	0.14			–	–	–	––	
Orally supplied	8 (2585)	52%	0.88 [0.72, 1.06]	0.18	0.28		8 (2585)	69%	0.10 [−0.48, 0.67]	0.75	–
**DHA+EPA dose**											
<1.8 g/d	4 (464)	75%	0.68 [0.34, 1.35]	0.27			4 (464)	65%	−0.03 [−1.04, 0.97]	0.95	
≥ 1.8g/d	5 (2223)	0%	0.93 [0.83, 1.05]	0.24	0.37		4 (2121)	74%	0.26 [−0.54, 1.05]	0.53	0.66
**EPA dose**											
<1 g/d	4 (464)	75%	0.68 [0.34, 1.35]	0.27			4 (464)	65%	−0.03 [−1.04, 0.97]	0.95	
≥ 1 g/d	5 (2223)	0%	0.93 [0.83, 1.05]	0.24	0.37		4 (2121)	74%	0.26 [−0.54, 1.05]	0.53	0.66
**DHA dose**											
>1 g/d	5 (657)	8%	0.63 [0.48, 0.83]	<0.001			4 (555)	0%	−0.65 [−1.21, −0.08]	0.02	
≤1 g/d	4 (2030)	0%	1.00 [0.89, 1.13]	0.99	0.002		4 (2030)	77%	0.58 [−0.20, 1.37]	0.15	0.01
**EPA/DHA**											
≥ 1.2	5 (2224)	2%	0.97 [0.86, 1.09]	0.59			5 (2224)	73%	0.44 [−0.31, 1.20]	0.25	
<1.2	4 (463)	0%	0.50 [0.34, 0.74]	<0.001	0.001		3 (361)	0%	−0.61 [−1.19, −0.03]	0.04	0.03
**POAF incidence in controls**											
<33%	5 (2062)	39%	0.83 [0.63, 1.09]	0.17			4 (1960)	72%	0.28 [−0.38, 0.94]	0.41	
≥ 33%	4 (625)	68%	0.87 [0.62, 1.21]	0.40	0.84		4 (625)	67%	−0.13 [−1.42, 1.16]	0.84	0.58
**Jadad Scores**											
≤3	6 (874)	51%	0.72 [0.52, 1.00]	0.05			5 (727)	77%	−0.01 [−0.95, 0.93]	0.98	
>3	3 (1813)	50%	0.97 [0.77, 1.22]	0.79	0.11		3 (1813)	61%	0.24 [−0.94, 1.42]	0.69	0.74

1 p values for subgroup effects.

2 p values for subgroup interaction.

POAF, post-operative atrial fibrillation; LOH, length of hospitalization; RR, relative risk; WMD, weighed mean difference; HTN, hypertension; DM, diabetes mellitus; COPD, chronic obstructive pulmonary disease; AF, atrial fibrillation; LVEF, left ventricular ejection fraction; LAD, left atrial dimension; ACEI, angiotensin converting enzyme inhibitors; ARB, angiotensin II receptor blockers; DHA, ducosahexaenoic acid; EPA, eicosapentaenoic acid.

### Effects of fish oil on the length of hospitalization after surgery

Eight comparisons [18], [20], [21], [22], [23], [24], [25], of which fish oil was all supplied orally, including 1285 patients in the fish oil groups and 1300 patients in the control groups, investigated the influence of fish oil supplementation on LOH after cardiac surgery. Overall, the pooled analysis indicated that fish oil did not have a significant influence on LOH (WMD  = 0.10 days, 95% CI −0.48 to 0.67 days, p = 0.75; **Figure** **3B**), although significant heterogeneity was found (I^2^ = 69%, p = 0.002). Results of meta-regression analyses suggested that mean dose of DHA was positively related to the reduction of LOH after fish oil supplementation (coefficient  = −2.1, p = 0.04; **Table** **4** and **Figure** **4B**). Similarly, results of subgroup analyses also suggested that fish oil may significantly reduce LOH after surgery in patients taking DHA >1g/d [18], [22], [23] (WMD  = −0.65 days, 95% CI −1.21 to −0.08 days, p = 0.02; **Figure** **3B** and **Table** **5**), but did not in patients taking a lower dose of DHA [20], [21], [24], [25]. Consistently, lower ratio of EPA to DHA (<1.2) was also associated with a significant reduction of LOH after surgery [18], [23] (**Table** **5**). Of note, the two studies with a lower ratio of EPA to DHA were all of an open-label design. Besides, the results of subgroup analyses suggested that preoperative use of β-blockers may influence the effect of fish oil on LOH. It seemed that fish oil may increase LOH in studies which included more patients taking β-blockers (≥70%, **Table** **5**), although meta-regression results did not support baseline β-blockers usage as a significant modifier of the effects of fish oil on LOH after surgery (**Table** **4**).

### Effects of fish oil supplementation on other outcomes

**Figure 5:**
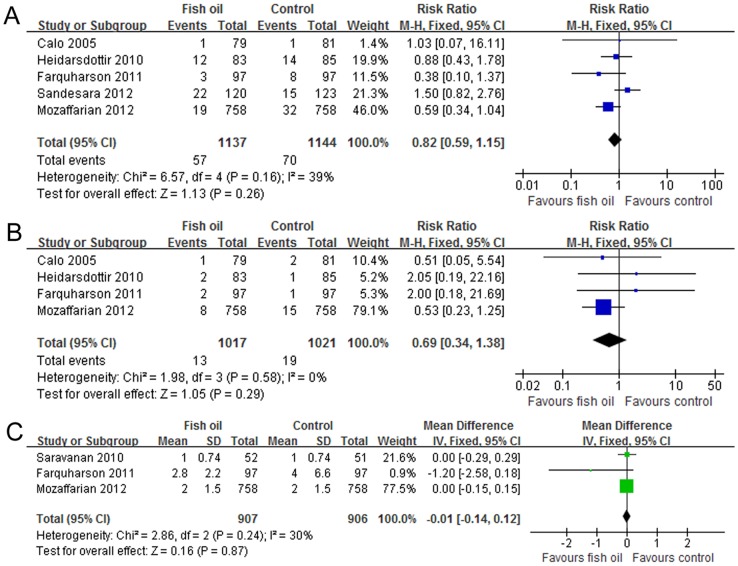
Forest plots from meta-analyses for the effects of fish oil supplementation on incidence of major bleeding (A), perioperative mortality (B) and length of ICU stay after cardiac surgery (C). The effect size of each study is proportional to the statistical weight. The diamond indicates the overall summary estimate for the analysis; the width of the diamond represents the 95% CI. ICU, intensive care unit; M-H, Mantel-Haenszel; IV, inverse variance; CI, confidence interval; SD, standard deviation.

Major bleeding was defined as bleeding more than 3 L through chest tube drain [22], needing transfusion [24], or needing reexploration or reoperation [18], [21], [24], [25] . By pooling these studies [18], [21], [22], [24], [25], we found no significant influence of fish oil on the incidence of major bleeding (I^2^ = 39%, fixed-effect model: RR = 0.82, 95% CI 0.59 to 1.55, p = 0.26; random-effect model: RR = 0.83, 95% CI 0.51 to 1.35, p = 0.45; **Figure** **5A**). Limited studies suggested that fish oil supplementation did not seem to affect perioperative mortality [18], [21], [22], [25] (**Figure** **5B** **)** or postoperative ICU stay [20], [22], [25] (**Figure** **5C**) significantly.

### Publication biases

**Figure 6:**
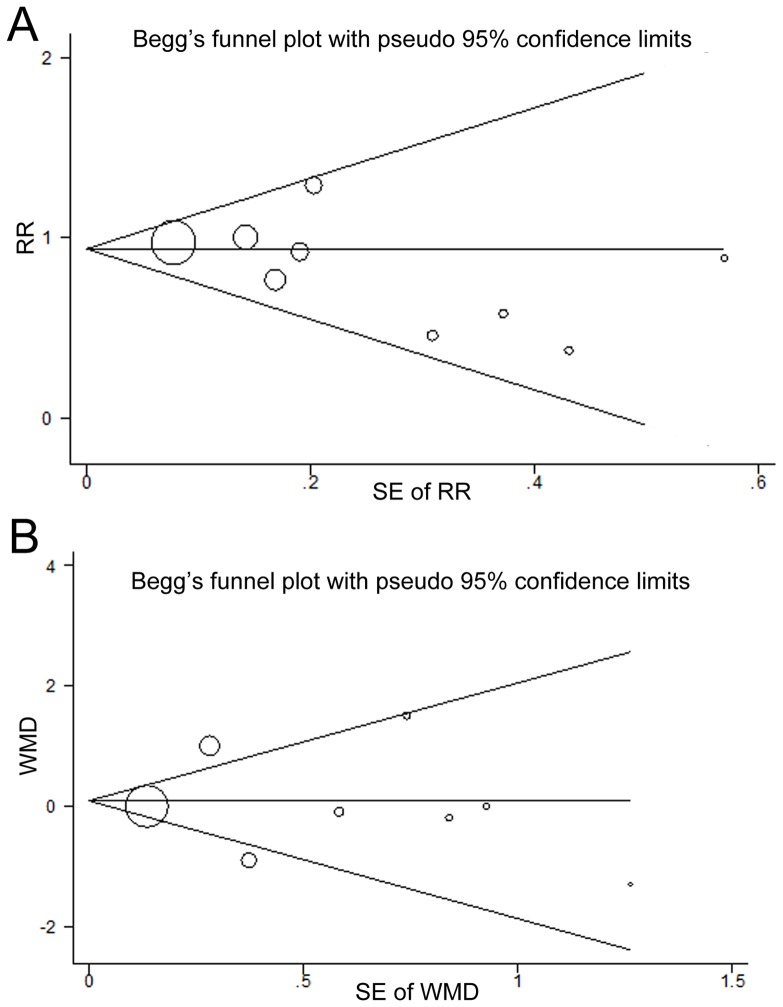
Funnel plots (with pseudo 95% CIs) of all the individual studies in the meta-analyses for the effects of fish oil supplementation on POAF incidence (A) and LOH after cardiac surgery (B). POAF, postoperative atrial fibrillation; LOH, length of hospitalization; RR, risk ratio; WMD, weighed mean difference, SE, standard error; CI, confidence interval.

Funnel plots (**Figure** **6A and 6B**) and Egger's regression asymmetry tests of the included studies did not suggest significant publication biases for the effects of fish oil supplementation on the incidence of POAF (Egger's test p = 0.18) or LOH after surgery (Egger's test p = 0.79). For the other endpoints of the current analyses, we were unable to estimate the publication biases due to limited number of studies included.

## Discussion

In this study, by pooling the results of available randomized controlled trials, we did not find that perioperative fish oil supplementation could significantly reduce the incidence of POAF or LOH after cardiac surgery. However, significant heterogeneity existed among these studies. Of note, results of meta-regression and subgroup analyses suggested that relative amount of DHA and EPA seemed to be a potential modifier to the effects of fish oil on POAF and LOH. Specifically, fish oil may reduce the POAF incidence and LOH in studies with supplements of mean DHA dose over 1.0 g/d or ratio of EPA to DHA <1.2, but did not in studies with supplements of a lower dose of DHA or a higher ratio of EPA to DHA. In addition, current evidence also did not support fish oil supplementation could significantly affect perioperative mortality, incidence of major bleeding, or the length of ICU stay after cardiac surgery.

Early prospective cohort study indicated that fish consumption with high n-3 PUFAs is associated with a lower incidence of AF in humans after follow-up for 12 years [32], suggesting that fish oil supplementation may be applied as a strategy for the long-term primary prevention of AF. However, some similar dietary studies [33], [34], [35], [36] published later failed to show an association between increased fish or n-3 PUFAs consumption and decreased risk of incident AF. On the other hand, many small-scaled controlled trials and meta-analyses observed that supplementation with fish oil could favorably affect some pathophysiological processes which contribute to the pathogenesis of AF, including inflammation [37], [38], endothelial dysfunction [39], ventricular remodeling [40], and possibly autonomic disturbance [41], [42]. These findings promoted related studies in humans to investigate whether supplementation with fish oil can prevent the incidence of POAF. Although the results of some early studies [18], [19] and meta-analysis [43] seemed to be promising, a recent published large-scale trial [25] including 1516 such patients failed to support the potential preventative role of perioperative fish oil supplementation for the incidence of POAF. Results of our meta-analysis, by including these published randomized controlled trials, also did not show a significant association between fish oil supplementation and reduced POAF incidence, though considerable heterogeneity was detected among the studies. Therefore, currently there is no sufficient evidence to recommend fish oil as a preventative measure for POAF.

Interestingly, we found that relative amount of DHA and EPA in the supplements seemed to be a significant modifier to the effect of fish oil supplementation on POAF, which may be an important source of heterogeneity among the included studies. Particularly, supplements with a relatively higher amount of DHA (>1g/d) seemed to relate to a significant decreased risk of POAF and LOH after surgery, while did not for supplements with lower amount of DHA, suggesting that DHA may be the determinant for the potential preventative effect of fish oil for POAF. These findings are consistent with the results of recent cross-sectional [44] and cohort studies [45], [46], which suggested that lower serum content of DHA but not EPA was independently associated with increased risk of AF in humans. Although the exact mechanisms underlying the possible different effects of DHA and EPA on POAF are unknown, there is some evidence from studies in animals and humans [47], [48] which indicated that compare to EPA, DHA can favorably influence some physiological processes involved in AF pathogenesis in a more effective way, such as anti-inflammation and reduction of heart rate. Besides, previous studies demonstrated that although supplying with pure DHA can also raise serum and tissue levels of EPA, the reverse cannot be achieved [47]. Furthermore, DHA is more abundant than EPA in the myocardium, also reflecting that DHA may be more important for the normal heart function [11]. However, our findings that supplements with higher DHA (>1g/d) was associated with reduced POAF incidence and LOH after surgery should be interpreted with caution because 3 of the five comparisons included in the subgroup were of open-label design, and the numbers of the patients included in the subgroup (n = 657) are only a small part of the patients included in the whole analysis (about 24.5%).

Previous studies suggested that pretreatment of medications such as ACEIs/ARBs [49], statins [50] and β-blockers [51] may reduce the incidence of POAF, and the incidence of POAF may also decrease if off-pump cardiac surgeries were applied [52]. However, results of meta-regression and subgroup analyses seemed not to support that the above factors may influence the effects of fish oil supplementation on POAF. Of note, because we do not have individual patient data of the included studies, these meta-regression and subgroup analyses were generally based on the proportions of the included patients in each study who were pretreated with these medications or performed with off-pump surgeries. Obviously, results of these subgroup analyses should be interpreted cautiously and future studies are needed to evaluate whether fish oil supplementation could reduce the incidence of POAF in those who were not pretreated with these medications or in those who received on-pump cardiac surgeries.

Results of our study also indicated that fish oil supplementation did not significantly influence the perioperative mortality, incidence of major bleeding or the length of ICU stay after surgery. These results, together with the facts that none of the included study reported serious adverse events which were deemed to be caused by fish oil supplementation, suggested that fish oil is safe and well tolerated by these patients.

Several potential limitations should be concerned regarding the present meta-analyses. Firstly, the included studies were different in study scale and design, patient characteristics, concurrent therapy, surgical performance and regimens of fish oil supplementation, which may contribute to the heterogeneity among the studies. Besides, these studies also lack homogeneity in both method of postoperative monitoring and in the definition of POAF, which may make the interpretation of the results difficult. Moreover, the baseline status of serum contents of n-3 PUFAs before the trials was not collected in some studies, which may influence the effect of fish oil supplementation on POAF. Finally, as described previously, some relatively low-quality studies were included, and the numbers of studies and patients included for some subgroup analysis were small, so interpretation of the subgroup results should be with caution.

In conclusion, results of our meta-analysis did not support the preventative effects of perioperative fish oil supplementation for the incidence of POAF, although fish oil can be safe and well tolerated by these patients and did not significantly affect the perioperative mortality, incidence of major bleeding, and hospital and ICU stays. In addition, relative amount of DHA and EPA seemed to be an important modifier of the effect of fish oil for POAF. Supplements with DHA >1 g/d may be associate with a significant reduced risk of POAF, while did not for supplements with lower dose of DHA. Results of our study indicated that DHA and EPA may be different for prevention of POAF. Although these findings need to be confirmed in future clinical trials and experimental studies, our study highlighted the fact that the relative amounts of DHA and EPA in fish oil is important for at least some of its cardioprotective effects, such as prevention of POAF.

## Supporting Information

Checklist S1 **PRISMA Checklist.**
(DOC)
